# Risk Factors for Wound Complications and Hernia Recurrence in Abdominal Wall Reconstruction: A Single-Institution Retrospective Study

**DOI:** 10.7759/cureus.88565

**Published:** 2025-07-23

**Authors:** Mohamed Wael Ahmed, Yousra Elshoura, Ali Mohamedahmed, Sadhasivam Ramasamy, Georgios Kakaniaris, Zbigniew Muras, Himaz Marzook

**Affiliations:** 1 General Surgery, Queen's Hospital Burton, University Hospitals of Derby and Burton NHS Trust, Burton-on-Trent, GBR; 2 General Surgery, University Hospitals of Coventry and Warwickshire NHS Trust, Coventry, GBR; 3 General Surgery, The Royal Wolverhampton NHS Trust, Wolverhampton, GBR

**Keywords:** abdominal wall reconstruction, body mass index, hernia recurrence, incisional hernia, rives-stoppa technique, surgical site infection, wound complications

## Abstract

Introduction: Incisional hernia (IH) remains a frequent and challenging postoperative complication, often requiring complex abdominal wall reconstruction (AWR). This study aimed to evaluate the outcomes of AWR and identify risk factors for wound complications and hernia recurrence at a district general hospital.

Methods: A retrospective cohort study was conducted at Queen's Hospital Burton, including 42 patients who underwent elective midline AWR between June 2017 and December 2023. Data on patient demographics, hernia characteristics, operative details, and postoperative outcomes were collected. Primary outcomes were hernia recurrence and wound complications. Secondary outcomes included hospital length of stay, postoperative ileus, fistula formation, and reoperation. Univariate statistical analysis was performed to identify predictors of wound complications; analysis for risk factors for recurrence was not feasible due to the low event rate.

Results: The mean patient age was 60.4 ± 12.5 years, with a mean BMI of 32.4 ± 5.0 kg/m². The most common repair technique was Rives-Stoppa, used in 28 (66%) patients. Hernia recurrence occurred in two (4.8%) patients, while wound complications were observed in 21 (50%) patients, predominantly seromas, which were noted in 12 (28.5%) patients. A BMI >35 kg/m² was significantly associated with wound complications (p=0.016). Other factors, including age, diabetes, smoking status, and hernia type, were not statistically significant predictors. The mean hospital stay was 5.4 ± 3.15 days, with a single postoperative mortality (2.4%).

Conclusion: AWR using the Rives-Stoppa technique with retrorectus mesh placement resulted in low recurrence rates and acceptable morbidity, although postoperative wound complications occurred in half of the patients. High BMI was a significant predictor of wound complications. These findings underscore the importance of tailored surgical planning and preoperative optimization in high-risk patients undergoing complex ventral hernia repair.

## Introduction

Incisional hernia (IH) represents a frequent postsurgical complication, with reported incidence rates varying widely but potentially affecting 10% to over 30% of patients following laparotomy [1]. The development of an IH significantly diminishes patient well-being, often leading to chronic pain, functional limitations, and a demonstrable negative impact on health-related quality of life [2]. The surgical management of IH presents considerable challenges, influenced by a confluence of factors such as patient-specific comorbidities (e.g., obesity, diabetes, smoking status), hernia characteristics (including defect size, location, loss of domain), and the complexity introduced by prior abdominal surgeries or previous failed hernia repairs [3]. Consequently, while surgical repair via abdominal wall reconstruction (AWR) is the standard approach, significant debate persists within the surgical community regarding optimal treatment strategies. Key areas of ongoing discussion include the relative merits of open versus minimally invasive (laparoscopic or robotic) surgical approaches, the specific indications for prosthetic mesh reinforcement versus primary suture repair, and the comparative effectiveness of various available mesh types (e.g., synthetic, biologic, bioabsorbable) and their ideal anatomical placement [4]. Notwithstanding these controversies, a substantial body of evidence supports the principle that the placement of prosthetic mesh in a retromuscular (sublay) position, as a technique, is associated with reduced rates of long-term hernia recurrence compared to alternative placements, such as onlay techniques [5]. This single-center study aimed to identify patient-specific risk factors for postoperative morbidity by analyzing outcomes from a cohort treated at a district general hospital, which helps minimize the confounding effects of surgeon variability and perioperative protocols.

## Materials and methods

Study design and patient population

This study was conducted as a retrospective observational cohort analysis. Institutional surgical databases were used to identify all consecutive patients who underwent AWR at Queen's Hospital Burton between June 2017 and December 2023.

Eligible patients (n=42) were those who underwent elective AWR within this timeframe and had a minimum of 12 months of postoperative follow-up, allowing sufficient time for outcome assessment. The primary objective of the study was to evaluate the rates of hernia recurrence and wound-related complications, specifically surgical site infection (SSI), wound dehiscence, and seroma formation. Hernia recurrence was diagnosed based on clinical examination during outpatient follow-up every four to six months and confirmed with a CT scan when there was clinical suspicion. Secondary objectives included assessing the length of hospital stay, the occurrence of enterocutaneous fistula, the development of postoperative ileus, and unplanned reoperations. Data were extracted retrospectively from the hospital’s electronic medical record system (Meditech; Medical Information Technology Inc., USA).

Patients were excluded if they presented with lateral abdominal wall hernias, parastomal hernias, active infections at the time of surgery, strangulated or irreducible hernias, or if they required emergency surgical intervention. Lateral hernias were classified using the European Hernia Society (EHS) system, which refers to defects located lateral to the rectus sheath, categorized as L1 to L4 [6]. Patients with less than 12 months of documented postoperative follow-up or whose records were incomplete were excluded from the final analysis.

Only midline hernias, classified as M1 to M5 under the EHS system, were included. This standardized classification allowed for anatomical consistency across the study population and facilitated the comparison of outcomes based on hernia location [6].

Data collection

Data were retrospectively collected from the Meditech electronic records system. The information gathered included patient demographics such as age, sex, smoking status, body mass index (BMI), and relevant comorbidities, including diabetes mellitus, cardiovascular disease, immunosuppression, and history of previous abdominal surgery. Details of hernia characteristics, including location, size, primary versus incisional type, and recurrence status, were recorded according to the EHS classification.

Operative details were extracted, including the type of surgical approach (open or laparoscopic), intraoperative bowel injury, conversion to open surgery, whether component separation was performed, the use and type of mesh (synthetic or biologic), and the position of mesh placement. Postoperative outcomes were recorded and included the presence of SSI, wound dehiscence, seroma formation, hernia recurrence, enterocutaneous fistula, postoperative ileus, length of hospital stay, and whether a reoperation was required. The data collection period concluded after adequate patient follow-up was recorded in the system, up to the study's end date.

Outcomes and variables

The primary outcomes evaluated were hernia recurrence rates and the incidence of wound-related complications during the follow-up period. These complications were defined as SSI, wound dehiscence, and seroma formation. While hernia recurrence was a primary outcome, a predictor analysis for this outcome was not performed due to the low number of events (n=2), which precluded meaningful statistical evaluation. Secondary outcomes included the length of hospital stay, the occurrence of enterocutaneous fistula, postoperative ileus, need for reoperation, 30-day hospital readmission, and postoperative respiratory complications. Potential predictor variables included patient demographics and relevant operative details gathered during data collection.

Statistical analyses 

Descriptive statistics were employed to summarize the baseline characteristics of the study population, including calculation of means and standard deviations for continuous variables and frequencies with percentages for categorical variables. Univariate analysis was first conducted to explore associations between predictor variables and the primary and secondary outcomes, using appropriate statistical tests. Multivariate analysis was not feasible due to the limited sample size (n=42), which would not support a robust regression model. Statistical significance was set at p<0.05. Given the exploratory nature of the subgroup analysis, no correction for multiple comparisons was applied. Furthermore, a post hoc power estimate was not performed; the small sample size is acknowledged as a key limitation that may prevent the detection of smaller yet potentially clinically significant associations. All analyses were carried out using IBM SPSS Statistics for Windows, v27.0 (IBM Corp., Armonk, USA).

## Results

Baseline characters

A review of the included 42 patient records confirmed a complete dataset with no missing values for the key demographic, operative, or outcome variables analyzed. The study population mean age was 60.4 ± 12.5 years, with a slight female predominance (n=24, 57%). The mean BMI for the included patients was 32.4 ± 5.0 kg/m². Notable comorbidities included diabetes (n=4, 9.5%). A considerable portion of the patients were smokers (n=20, 47.6%). The mean postoperative follow-up for the cohort was 34.0 ± 15.2 months. The mean hernia defect size was 10.9 ± 4.8 cm, with the majority being incisional or recurrent (n=34, 81.0%). Surgical repairs primarily involved Rives-Stoppa (n=21, 50%) or transversus abdominis release (TAR) (n=10, 24%), with combined or other techniques used in the remainder. The baseline characters summary is mentioned in Table 1.

**Table 1:** Baseline characters TAR: transversus abdominis release; ACS: anterior component separation

Baseline characters		N=42	SD / %
Age (in years) (mean)		60.36	(±12.5)
Gender (n)	Male	18	43%
	Female	24	57%
BMI (in kg/m²) (mean)		32.4	(±5)
Diabetes (n)		4	9.52%
Smoker (n)		20	47.62%
Defect size (in cm) (mean)		10.9	(±4.8)
Type (n)	Incisional	16	38%
	Recurrent	18	43%
Repair (n)	Rives-Stoppa	21	50%
	TAR	10	24%
	Rives-Stoppa + ACS	4	9%
	Rives-Stoppa + TAR	3	7%
	Other	4	10%

Primary outcomes

Hernia recurrence was observed in two patients (4.8%). Wound complications occurred in 21 patients (50.0%), with the most common being seroma (n=12), followed by infection (n=5), hematoma (n=3), and fistula (n=1). Table 2 details the primary outcomes.

**Table 2:** Primary outcomes

Primary outcomes		N=42	%
Recurrence (n)		2	4.8
Wound complication (n)	Infection	5	11.9
	Hematoma	3	7
	Seroma	12	28.5
	Fistula	1	2

Secondary outcomes

Intraoperative iatrogenic bowel injury occurred in two patients (4.8%). One of these cases subsequently led to a postoperative leak, reoperation, and mortality from sepsis (representing a 2.4% mortality rate for the cohort). Reoperation related to the primary procedure was required in four patients (9.5%), with three patients reoperated due to wound infection. Other general complications included postoperative ileus in five patients (11.9%) and a respiratory complication in one patient (2.4%). The mean length of hospital stay was 5.4 ± 3.15 days. Five patients (11.9%) required readmission within 30 days of being discharged. A summary of secondary outcomes is presented in Table 3.

**Table 3:** Secondary outcomes

Secondary outcomes		N=42	SD / %
General complications (n)	Mortality	1	2.4
	Ileus	5	11.9
	Respiratory	1	2.3
Length of hospital stay (in days) (mean)		5.4	(±3.15)
Readmission (n)		5	11.9
Reoperation (n)		4	9.5

Univariate subgroup analysis exploring factors associated with wound complications is presented in Table 4. A statistically significant association was found between BMI category and the incidence of wound complications (p=0.016), primarily driven by a higher complication rate in patients with a BMI >35 kg/m². Recurrent hernia type was also significantly associated with wound complications compared to primary hernia (p=0.03). Other factors, including mean patient age (p=0.36), diabetes status (p=1.0), smoking status (p=1.0), and the type of surgical repair performed (Rives-Stoppa vs. TAR vs. other, p=0.3), did not demonstrate a statistically significant association with wound complications in this cohort. A trend nearing statistical significance was observed for mean defect size (p=0.059), suggesting that larger defects may potentially be linked to an increased risk of wound complications.

**Table 4:** Subgroup analysis for wound complications Statistical significance was set at p<0.05. (*) indicates p<0.05. TAR: transversus abdominis release

Subgroup		Yes (N=21)	No (N=21)	p-value
Age		62 (±14)	58 (±12)	0.36
BMI	<25	0	1	0.016*
	25-30	6	12	
	31-35	1	4	
	>35	14	4	
Diabetes		2	2	1
Smoker		9	10	1
Defect size		13 (±2)	10 (±5)	0.059
Recurrent		11	7	0.03*
Repair	Rives-Stoppa	8	13	0.3
	TAR	6	4	
	Other	7	4	

## Discussion

This retrospective cohort study analyzed the outcomes of 42 consecutive patients who underwent AWR for ventral hernias, prioritizing the Rives-Stoppa technique with retrorectus mesh placement over six years (2017-2023). This surgical approach was selected for its efficacy in managing large or complex hernia defects.

The Rives-Stoppa repair promotes tension-free fascial approximation, a critical factor in minimizing recurrence risk. Placement of prosthetic mesh within the retromuscular space leverages intra-abdominal pressure to distribute forces evenly across the reconstruction, thereby enhancing stability. This principle is consistent with prior reports demonstrating low recurrence rates; for example, recent studies continue to report favorable outcomes, with recurrence rates as low as 3.1% in some series utilizing modern mesh materials with this technique [7]. The low recurrence rate of two (4.8%) observed in the present series further corroborates the effectiveness of the Rives-Stoppa repair. This finding aligns closely with a large meta-analysis by Hartog et al. [8] encompassing 12,440 patients, which reported a 4.1% hernia recurrence within 24 months.

Retrorectus (sublay) mesh placement, central to the Rives-Stoppa technique, enhances tissue integration and fibrovascular growth, thereby improving repair stability while reducing complications such as visceral erosion and SSIs compared to onlay positioning [5]. The sublay position also optimizes the distribution of intra-abdominal forces, contributing to lower recurrence rates [9]. In our study, this approach demonstrated effectiveness for defects exceeding 10 cm in diameter, which were present in 20 (48%) cases, reinforcing its utility in complex reconstructions involving substantial fascial defects. This technique proved suitable for the challenging cases within this cohort, including recurrent hernias, which comprised 18 (42%) of the operative procedures, without an apparent increase in complication rates relative to the complexity addressed.

Conversely, other mesh positioning techniques are associated with higher risks of complications. Onlay mesh placement is correlated with increased rates of seroma formation and SSI due to the mesh's superficial location and potentially higher recurrence rates compared to retrorectus placement [10]. Intraperitoneal onlay mesh (IPOM) placement, while utilized, carries inherent risks of adhesion formation and potential erosion into adjacent viscera. Systematic reviews have indicated advantages for retrorectus mesh placement over alternatives, including intraperitoneal options [11], thus supporting the rationale for its use in this patient cohort.

Alternative surgical strategies for IH repair possess specific limitations. The anterior component separation (ACS), while effective for achieving midline fascial closure in large defects, necessitates extensive tissue dissection, which may increase wound morbidity rates, particularly in populations with elevated BMI [12]. Laparoscopic ventral hernia repair offers the benefits of minimally invasive surgery, including reduced postoperative pain and shorter hospital stays. However, its suitability for large defects requiring substantial reinforcement may be limited, with some studies suggesting potentially lower long-term reliability than open techniques, such as Rives-Stoppa, for extensive hernias [13]. This study's predominance of open repair, used in 36 (86%) procedures, reflects patient selection biased toward complex defects where long-term durability was prioritized.

Wound-related complications were documented in 21 (50%) patients in this series. While substantial, this should be interpreted in the context of the high-risk patient population undergoing AWR. For comparison, a recent multicenter study in the Netherlands, evaluating open reconstructions with a polypropylene-reinforced tissue matrix in a similarly high-risk cohort, reported a wound complication rate of 78% [14]. A notable, statistically significant association was observed between the occurrence of complications and a BMI greater than 35 kg/m². This finding aligns with recent systematic reviews and meta-analyses that identify obesity as a significant independent risk factor for postoperative wound complications, potentially increasing the risk several-fold in abdominal and hernia surgery [15]. This is attributed to factors such as altered tissue perfusion and increased mechanical strain [16].

Additionally, patients undergoing repair for recurrent hernias inherently present increased technical challenges due to scar tissue, distorted anatomy, and potentially compromised tissue quality - factors known to elevate complication risk [17]. The present analysis identified recurrent hernia as another statistically significant predictor of wound complications (p=0.03), which correlates with the study by Karamanos et al. [18], who found in their univariate analysis that recurrent hernia repair was associated with a significantly higher probability of surgical site complications compared to primary repair. The complication rates observed reflect the inherent complexity of the patient population treated.

Although not reaching statistical significance, a trend was observed indicating that larger hernia defects may increase the risk of wound complications (p=0.059). This is clinically plausible, as larger defects require more extensive tissue dissection; however, this finding requires validation in a larger cohort.

Limitations of this study include its retrospective design, small sample size, and potential selection bias. The lack of long-term follow-up and quality-of-life assessments restricts the generalization of these findings. Furthermore, this study did not include a priori sample size calculation, which results in our subgroup analysis likely being underpowered. This makes it particularly challenging to draw firm conclusions about nonsignificant trends, which may be subject to a Type II statistical error. Future studies should consider a prospective cohort design with a larger sample size and the inclusion of patient-reported outcomes to evaluate functional recovery post-reconstruction.

## Conclusions

In summary, this retrospective study observed a low hernia recurrence rate of two cases (4.8%) within the cohort. While promising, these results should be interpreted with caution due to the small number of events and the absence of predictor analysis. The placement of mesh in the retrorectus position yielded superior results in terms of recurrence prevention. Regarding morbidity, postoperative wound complications occurred with significantly greater frequency among patients with a BMI >35 kg/m² and those presenting for recurrent hernia repair. A positive correlation was also established between wound complications and the length of hospitalization. These results emphasize the critical importance of tailoring the surgical approach, which must include robust preoperative optimisation to account for individual patient variables and the specific complexities of the hernia. Furthermore, this study provides additional evidence supporting retrorectus mesh placement as a robust standard for the surgical treatment of complex ventral hernias. 
